# Fast food increases postprandial cardiac workload in type 2 diabetes independent of pre-exercise: A pilot study

**DOI:** 10.1186/s12937-015-0069-1

**Published:** 2015-08-14

**Authors:** Siri Marte Hollekim-Strand, Vegard Malmo, Turid Follestad, Ulrik Wisløff, Charlotte Björk Ingul

**Affiliations:** 1 K.G. Jebsen Centre of Exercise in Medicine at Department of Circulation and Medical Imaging, Faculty of Medicine, Norwegian University of Science and Technology, PO Box 8905, 7491 Trondheim, Norway; 2 Department of Cardiology, St. Olavs Hospital, Trondheim, Norway; 3 Department of Public Health and General Practice, Faculty of Medicine, Norwegian University of Science and Technology, Trondheim, Norway

## Abstract

**Background:**

Type 2 diabetes aggravates the postprandial metabolic effects of food, which increase cardiovascular risk. We investigated the acute effects of fast food on postprandial left ventricular (LV) function and the potential effects of pre-exercise in type 2 diabetes individuals.

**Methods:**

We used a cross-over study including 10 type 2 diabetes individuals (7 male and 3 females; 53.4 ± 8.1 years; 28.3 ± 3.8 kg/m^2^; type 2 diabetes duration 3.1 ± 1.8 years) and 10 controls (7 male and 3 females; 52.8 ± 10.1 years; 28.5 ± 4.2 kg/m^2^) performing high intensity interval exercise (HIIE; 40 min, 4 × 4min intervals, 90–95 % HR_max_), moderate intensity exercise (MIE; 47 min, 70 % HR_max_) and no exercise (NE) in a random order 16–18 hours prior to fast-food ingestion. Baseline echocardiography, blood pressure and biochemical measurements were recorded prior to and 16–18 hours after exercise, and 30 minutes, 2 hours and 4 hours after fast food ingestion.

**Results:**

LV diastolic (peak early diastolic tissue velocity, peak early diastolic filling velocity), and systolic workload (global strain rate, peak systolic tissue velocity, rate pressure product) increased after consumption of fast food in both groups. In contrast to controls, the type 2 diabetes group had prolonged elevations in resting heart rate and indications of prolonged elevations in diastolic workload (peak early diastolic tissue velocity) as well as reduced systolic blood pressure after fast food consumption. No significant modifications due to exercise in the postprandial phase were seen in any group.

**Conclusions:**

Our findings indicate that fast-food induces greater and sustained overall cardiac workload in type 2 diabetes individuals versus body mass index and age matched controls; exercise 16–18 hours pre-meal has no acute effects to the postprandial phase.

**Trial registration:**

ClinicalTrials.gov: NCT01991769.

## Background

Most of the day is spent in the postprandial state and frequent ingestion of energy dense food, rich in processed carbohydrates, saturated fats and salt (fast-food), increases the risk of cardiometabolic diseases [1–3].

Type 2 diabetes aggravates the postprandial metabolic effects of food because it impairs the transport, delivery and/or storage of carbohydrates and fats [4]. Although type 2 diabetes is an accepted cause of heart failure [5] and approximately 50 % of asymptomatic individuals with well-controlled type 2 diabetes show signs of diastolic dysfunction [6], little is known about the acute effects of food in the view of cardiac function in this population.

Endothelial function is impaired after fast food ingestion, which is related to increases in circulating glucose, triglycerides and/or elevated oxidative stress [7, 8]. However little is known about the acute consequence of excessive elevations in circulating glucose and/or triglycerides after a meal on cardiac function in type 2 diabetes.

Although few studies have investigated the acute effects of fast food on cardiac function, it is observed that food-induced elevation of circulating glucose and oxidative stress reduce diastolic function in insulin-treated type 2 diabetes patients [9], and that acute elevations in circulating triglycerides yield compensatory increases in systolic function of the left ventricle in healthy individuals [10].

Chronic exercise improves cardiac function [6] and even single bouts of exercise can improve endothelial function, triglycerides and oxidative stress in healthy and reduce postprandial glucose elevations in type 2 diabetes individuals [8, 11, 12]. Exercise 16–18 hours pre-meal has previously shown to induce improvements in total antioxidant status (TAS) and endothelial function, rather than reduce the circulating glucose or triglycerides [8]. In this setting, high intensity interval exercise (HIIE) was more effective in improving postprandial endothelial function compared to moderate intensity exercise (MIE) [8].

No study has investigated whether fast food ingestion induces an acute increase in cardiac workload, or whether this may be modified by pre-exercise as observed for endothelial function [8].

The purpose of this study was thus to explore whether a single fast food meal affects left ventricular (LV) diastolic and systolic function in the four hour postprandial phase, and whether exercise (HIIE or MIE) 16–18 hours prior to a single fast food meal could affect LV function, resting heart rate, blood pressure and/or other biochemical measures in type 2 diabetes patients.

## Methods

### Study participants

Type 2 diabetes individuals and healthy controls were recruited through a local newspaper and from advertisement at St. Olav’s University hospital, Trondheim, Norway. The study was performed from February to June 2012.

The inclusion criteria included: age 40 to 65 years and type 2 diabetes within the past 10 years with no use of insulin. Exclusion criteria included: known cardiovascular or lung disease, uncontrolled hypertension, orthopaedic or neurological restrictions, body mass index (BMI) >35 kg/m^2^, pregnancy, inability to exercise, smoking, drug or alcohol abuse, planned surgery during the trial period, serious eating- and/or personality disorders, reluctance to sign informed consent form, or more physical activity than today’s recommendations [13].

Ten type 2 diabetes individuals and 10 healthy age and BMI matched controls were included. The protocol was approved by the Regional Committee for Medical and Health Research Ethics and registered in the Clinical Trials Registry (ClinicalTrials.gov identifier: NCT01991769). Informed consent was obtained from all participants.

### Design

**Fig. 1:**
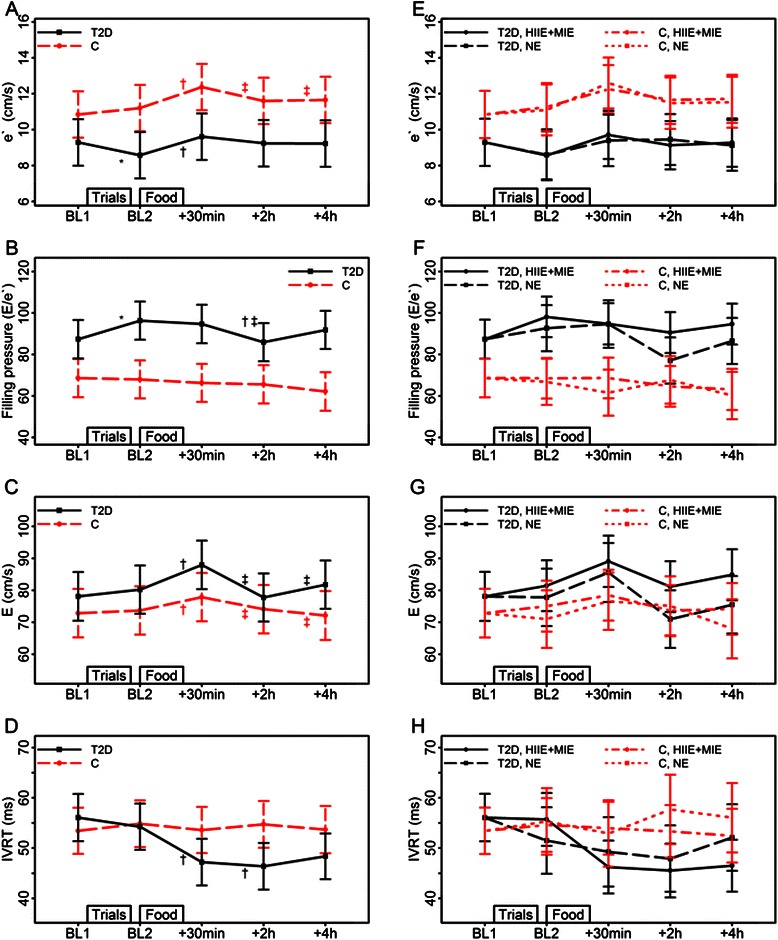
Effects of fast food (*left* panel; all trials combined) and exercise (*right* panel; high intensity interval exercise +moderate intensity exercise vs. no exercise) on left ventricular diastolic function. Abbreviations: BL, baseline; C, control group; e’, peak early diastolic tissue Doppler velocity; E/e’ , filling pressure; E, peak early filling velocity; HIIE, high intensity interval exercise; HIIE+MIE, exercise combined; IVRT, isovolumic relaxation time; MIE, moderate intensity exercise; NE, no exercise; T2D, type 2 diabetes group. Estimated means and 95 % CIs from LMMs with the factors time, group and their interaction (left panel, figures **a**-**d**), and with the factors time, group, trial and their interactions (right panel, figures E-H). In the left panel significant (*p* < 0.01) time differences are indicated by *(from BL1), † (from BL2), ‡ (from food +30 min) and § (from food +2 h). For peak early filling velocity (**e**) there is no significant time and group interaction, and the indicated significant time differences refer to the main effect of time for both groups

**Fig. 2:**
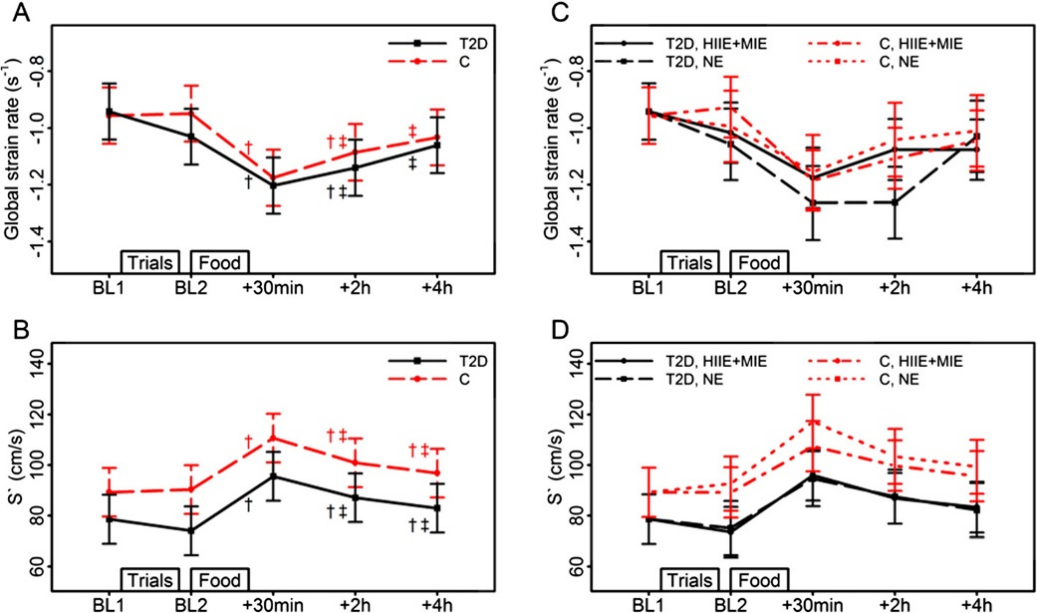
Effects of fast food (*left* panel; all trials combined) and exercise (*right* panel; high intensity interval exercise +moderate intensity exercise vs. no exercise) on left ventricular systolic function. Abbreviations: BL, baseline; C, control group; HIIE, high intensity exercise; HIIE+MIE, exercise combined; MIE, moderate intensity exercise; NE, no exercise; S' , peak systolic tissue Doppler velocity; T2D, type 2 diabetes group. Estimated means and 95 % CIs from LMMs with the factors time, group and their interaction (*left* panel, figures **a**-**b**), and with the factors time, group, trial and their interactions (right panel, figures **c**-**d**). In the left panel significant (*p* < 0.01) time differences are indicated by *(from BL1), † (from BL2), ‡ (from food +30 min) and § (from food +2 h). For S' and global strain rate there is no significant time and group interaction, and the indicated significant time differences refer to the main effect of time for both groups

**Fig. 3:**
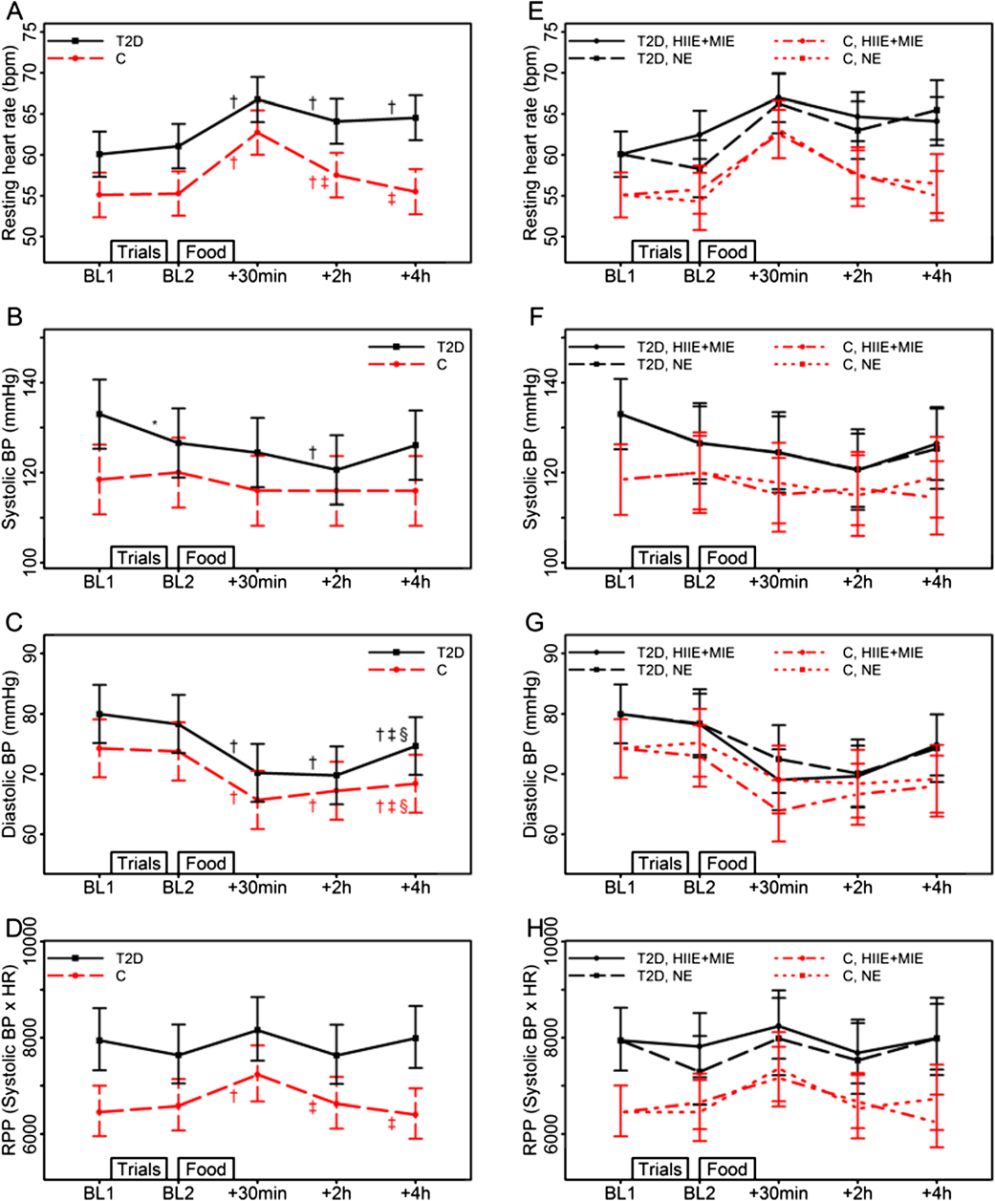
Effects of fast food (*left* panel; all trials combined) and exercise (right panel; high intensity interval exercise+moderate intensity exercise vs. no exercise) on resting heart rate and blood pressure. Abbreviations: BL, baseline; BP, blood pressure; C, control group; HIIE, high intensity exercise; HIIE+MIE, exercise combined; HR, resting heart rate; MIE; moderate intensity exercise; NE, no exercise; RPP, rate pressure product; T2D, type 2 diabetes group. Estimated means and 95 % CIs from LMMs with the factors time, group and their interaction (*left* panel, figures **a**-**d**), and with the factors time, group, trial and their interactions (*right* panel, figures **e**-**h**). In the left panel significant (*p* < 0.01) time differences are indicated by *(from BL1), † (from BL2), ‡ (from food +30 min) and § (from food +2 h). For diastolic BP there is no significant time and group interaction, and the indicated significant time differences refer to the main effect of time for both groups. For RPP, the means and CIs are shown as back-transformed values, computed by direct exponentiation of the means and CIs from the LMM based on log-transformed data

**Fig. 4:**
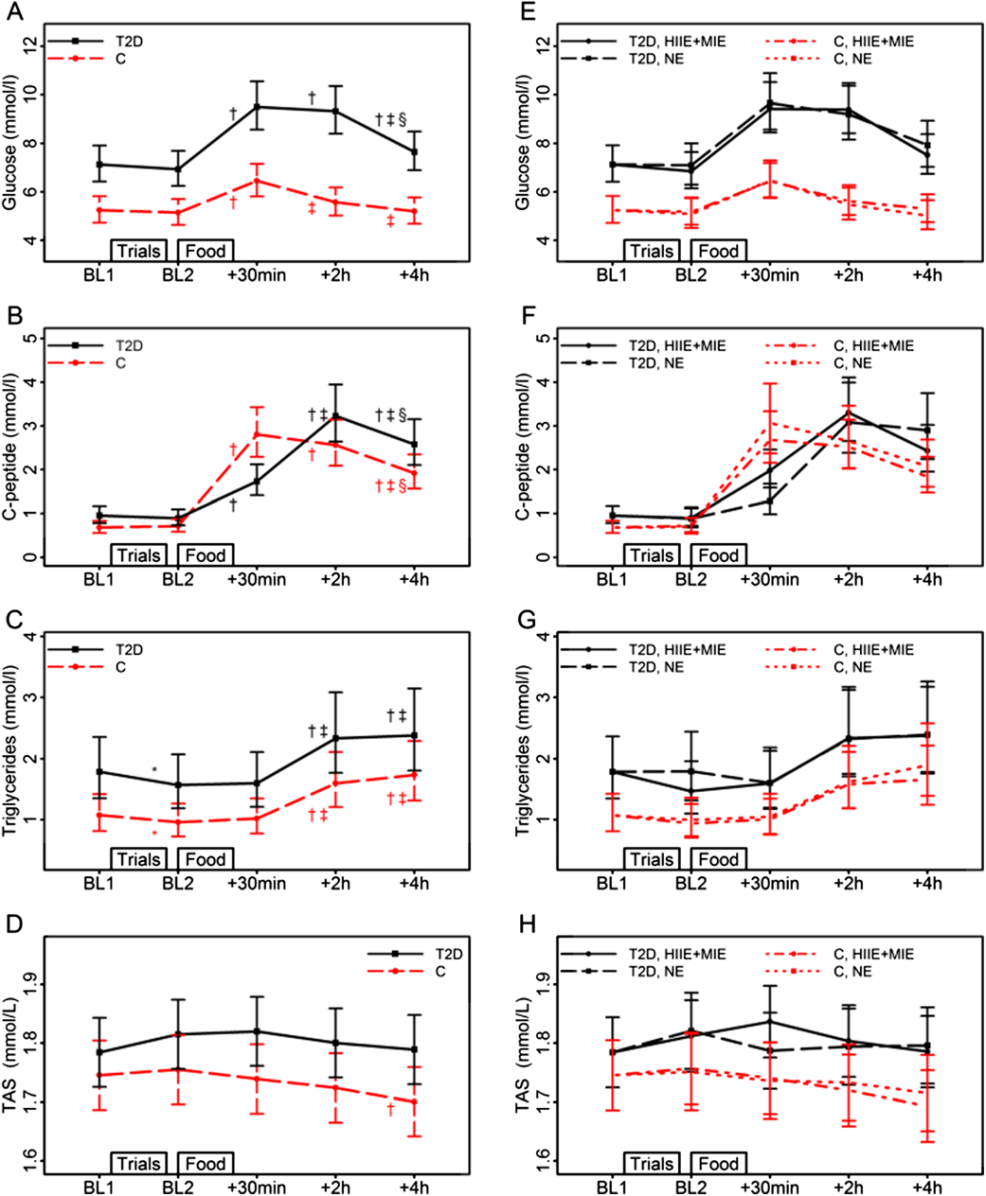
Effects of fast food (*left* panel, trials combined) and exercise (*right* panel; high intensity interval exercise+moderate intensity exercise vs. no exercise) on blood glucose, C-peptide, triglycerides and total antioxidant status. Abbreviations: BL, baseline; C, control group; HIIE,  high intensity exercise; HIIE+MIE, exercise combined; MIE, moderate intensity exercise; NE, no exercise; TAS, total antioxidant status; T2D, type 2 diabetes group. Estimated means and 95 % CIs from LMMs with the factors time, group and their interaction (*left* panel, figures **a**-**d**), and with the factors time, group, trial and their interactions (*right* panel, figures **e**-**h**). In the left panel significant (*p* < 0.01) time differences are indicated by *(from BL1), † (from BL2), ‡ (from food +30 min) and § (from food +2 h). For triglycerides there is no significant time and group interaction, and the indicated significant time differences refer to the main effect of time for both groups. Except for TAS, the means and CIs are shown as back-transformed values, computed by direct exponentiation of the means and CIs from the LMMs based on log-transformed data

A minimum of one week before the first trial, peak oxygen uptake (VO_2peak_) was assessed (Jaeger LE2000CE, Hochberg, Germany) as previously described [14]. Each of the 20 subjects participated in all trials (HIIE, MIE and no exercise (NE)), in a randomized order with a minimum of one week between trials. The timeline representing each trial is illustrated in Figs. 1, 2, 3 and 4. Baseline-1 measurements were performed on the day prior to fast-food ingestion in a resting (sedate behavior/refrained from exercise, ≥48 hours) and fasting (≥12 hours) state that abstained from caffeine, citrus and alcohol (16–18 hours). During the 16–18 hour period after HIIE, MIE or NE, the subjects were instructed to abstain from exercise, caffeine, citrus and alcohol and to report for baseline-2 in a fasting (≥12 hours) state the following morning. Before overnight fasting commenced, prior to all baseline-1 and baseline-2 measurements, subjects ingested a mixed meal at the same time and of the same content (same manufacturer, same amount) as they did before baseline-1 in their first trial. This meal was typically a standard Norwegian supper (i.e., whole grain sandwich with butter/margarine and meat/egg/fish/cheese and milk or water). Following baseline-2, fast food was ingested and measurements performed 30 minutes, 2 hours and 4 hours after the meal. The participants remained inactive in the laboratory after fast-food ingestion until end of the protocols.

### Outcome measures

The primary outcome measure was LV diastolic function measured as peak early diastolic tissue velocity (e’). Secondary outcome measures were LV late diastolic tissue velocity (a’), LV early diastolic filling velocity (E), LV late diastolic filling velocity (A), LV filling pressure (E/e’), E/A-ratio, deceleration time (DT), isovolumic relaxation time (IVRT), LV global strain and strain rate, LV peak systolic tissue velocity (S’) as well as resting heart rate, blood pressure, circulating glucose, triglycerides, total cholesterol, LDL-cholesterol, HDL-cholesterol, C-peptide, total antioxidant status (TAS) and high sensitive c-reactive protein (hs-CRP).

### Exercise training protocols

All exercise trials were supervised. HIIE was performed by walking or running on an inclined treadmill. Following 10 min warm up at approximately 70 % of maximal heart rate obtained at exercise testing (HR_max_), the HIIE-group performed four intervals at 90-95 % of HR_max_ with 3 minutes recovery periods between intervals at 70 % of HR_max_ and 5 minutes cool down; 40 min altogether. The MIE protocol consisted of 47 minutes exercise at 70 % of HR_max_ to achieve approximately similar total energy expenditure as for HIIE.

### Fast food

The fast food consisted of a vegetarian mozzarella pizza (Dr. Oetker); 335 g (874 kcal/ 3655 kJ), 83.4 g carbohydrates, 44.2 g fat, and 34.8 g protein. A previous study indicated that this pizza induces a marked postprandial increase in circulating glucose, triglycerides, C-peptide and a decrease in TAS as well as a transient impairment in endothelial function in healthy individuals [8].

### Clinical and laboratory examinations

#### Resting echocardiography

Three consecutive cycles in B-mode acquisitions (mean frame rate 53/sec) and color tissue Doppler imaging (TDI) (mean frame rate 159.9/sec) were recorded from the 3 apical views (four-chamber, two-chamber, and long- axis) and B-mode from the parasternal view. Measurements included E, A, IVRT and DT. Pulsed wave tissue Doppler velocities were measured at the four mitral annular sites in the four-chamber and two-chamber views. The mean of these points was used for S’ , e’ and a’ [15]. The ratio E/e’ was calculated as an estimate of LV filling pressure [16]. Global strain and strain rate were calculated from 2-dimensional strain echocardiography [17]. Measurements obtained in this study are in accordance with standard procedures recommended by the American Society of Echocardiography [18], and no subjects were excluded because of impaired echocardiographic image quality. Images were analyzed off line using EchoPAC version BT12 (GE Vingmed Ultrasound, Horten, Norway). The observer was blinded to group participation, trial and point in time during ultrasound analysis.

#### Resting heart rate and blood pressure

The lowest heart rate observed during echocardiography was defined as the resting heart rate. Upright blood pressure measurements were performed using Philips SureSigns V52 (Andover, Massachusetts, US). Before baseline-1 and baseline-2, participants rested in a sitting position for at least 10 minutes before measurements. Blood pressure at these time points was noted as the median of three recordings. At the remaining time points, upright blood pressure was measured only once. Rate pressure product (RPP; heart rate x systolic blood pressure) was calculated to determine myocardial workload.

#### Biochemical analysis

Blood was collected after blood pressure measurements and before echocardiography. Blood glucose, C-peptide, plasma triglycerides, total cholesterol, high density lipoprotein (HDL) and low density lipoprotein (LDL) and high-sensitive C-reactive protein (hs-CRP) were analyzed according to standard procedures at the St. Olavs Hospital (Trondheim) at all time-points; HbA_1c_ was measured at baseline-1. Blood glucose was measured using photometric hexokinase UV method (Roche Modular, Roche Diagnostics, Germany) and C-peptide was measured using chemiluminescence method (Immulite 2000, Siemens Medical Solutions, New Jersey, US). The blood lipids were measured using photometric, enzymatic colorimetric method (Roche Modular, Roche Diagnostics Germany). Hs-CRP was measured using Tina-quant CRPHS immunoturbidimetric assay (Roche Modular, Roche Diagnostics, Germany). HbA_1c_ was measured using TINIA (Turbidimetric Inhibition immunoassay) (Roche Cobas Integra 400 plus, Roche Diagnostics, Germany). Insulin sensitivity was calculated using the HOMA2 calculator (The Homeostasis Assessment Model, University of Oxford, UK). Total antioxidant status (TAS) was analyzed as previously described [19].

### Statistical methods

The statistical analysis was performed by linear mixed models (LMMs). Within-subject correlations were considered using a random intercept in the LMM. A full model included group (type 2 diabetes or controls), trial (HIIE, MIE, or NE), and time (baseline-1, baseline-2, and 30 minutes, 2 hours and 4 hours post-meal). Models with two levels of the trial factor (HIIE and MIE combined or NE) were also considered. Tests for overall effects of factors and factor interactions were done by likelihood ratio tests using significance level 0.05. Post hoc comparisons specified and tested appropriate linear combinations (contrasts) of the estimated model parameters for the selected models. In all models, the baseline means (baseline-1) for each group were restricted to be equal for the three exercise trials due to randomization to trials within each group [20]. Outcome variables not meeting the normal assumptions of the LMM were log transformed prior to the statistical analysis in cases where this transformation improved the approximation to the normal distribution. The analyses were performed in the R statistical package [21].

This study is explorative rather than confirmative, and thus we did not perform any formal adjustment for multiple testing. However, if nothing else is explicitly stated, the results presented and discussed here are statistically significant at p<0.01.

## Results

### Subject characteristics

Subject characteristics are reported in Table 1. Twenty participants completed all trials (HIIE, MIE, NE), and exercised according to prescribed exercise heart rates and followed instructions of sedentary behavior during the NE trial. No adverse events were reported. Since no statistically significant effects of pre-exercise were found, the results discussed below are from LMMs including the factors group and time.

**Table 1:** Subject characteristics

	Type 2 diabetes	Control	*p*
n	10	10	
Male/Female, n (%)	7/3 (70/30)	7/3 (70/30)	
Diabetes duration, years	3.1 ± 1.8	-	
Age, years	53.4 ± 8.1	52.8 ± 10.1	0.89
Body mass index, kg/m^2^	28.3 ± 3.8	28.5 ± 4.2	0.91
Waist circumference, cm	107.1 ± 27.5	104.4 ± 13.8	0.78
HbA_1c_, % (mmol/mol)	6.4 ± 1.0 (46.0 ± 7.0)	5.5 ± 0.2 (36.0 ± 1.5)	0.01
HOMA-ir	2.2 ± 0.7	1.7 ± 0.7	0.10
VO_2peak,_ ml/kg/min	38.8 ± 7.8	36.2 ± 8.8	0.51
VO_2peak_, L/min	3.39 ± 0.84	3.36 ± 0.92	0.93
Medical agents, n (%)			
Anti-diabetic	6 (60)	0 (0)	
Statins	1 (10)	1 (10)	
Anti-hypertension	6 (60)	1 (10)	

Data are means ± SD unless otherwise indicated

*Abbreviations*: *HbA1c* glycosylated hemoglobin, *HOMA-ir* homeostatic assessment model- insulin resistance, *VO*_*2peak*_ peak oxygen uptake

T-tests were used to test for differences between groups at baseline

### Effect of pre-exercise

Since no statistically significant effects of pre-exercise were found, the results discussed below are from LMMs including the factors group and time.

### Cardiac function

The LV diastolic responses to fast food are illustrated in Fig. 1a-d.

The type 2 diabetes group had an overall poorer diastolic function (e’) and higher filling pressure (E/e’) versus controls (Fig. 1a-b).

In general, diastolic workload increased (higher e’ , a’ , E and A) within 30 minutes after the meal in both groups. Subsequently, diastolic workload reversed towards baseline-2 levels, but in contrast to controls (*p* = 0.10), the type 2 diabetes group showed an indication of increased diastolic workload as measured by e’ that persisted 4 hours after the meal (*p* = 0.02). Late diastolic filling (a’) remained elevated 4 hours after fast food in both groups.

Filling pressure (E/e’) and isovolumic relaxation time (IVRT) were reduced within 2 hours after the meal in the type 2 diabetes group; this was not significantly different after fast food in the controls. The difference between groups in change from baseline-2 to 2 hours post-meal was almost significant for IVRT (*p* = 0.03) but not for filling pressure (*p* = 0.08). No effect of time was observed for E/A ratio because both E and A increased. Supernormal filling is associated with vigorous recoil of the ventricle during diastole with an increase of negative pressure in the ventricle and evacuation of blood from the atrium. This causes a high E wave, shortening of the IVRT and normal deceleration time. We found that the shortened IVRT and lack of changes to deceleration time might be due to the same mechanism.

Pre-exercise did not influence postprandial early diastolic velocity (e’) (Fig. 1e) or any other diastolic echocardiographic variables (Fig. 1f-h).

The LV systolic response to fast food are illustrated in Fig. 2a-b. In general, systolic workload (global strain rate and S’) increased 30 min after the meal in both groups. Systolic workload was subsequently reversed, but remained significantly elevated (global strain rate and S’) compared to baseline-2 after 4 hours in both groups (Fig. 2a-b).

Pre-exercise did not affect systolic function (Fig. 2c-d).

### Hemodynamic measurements

The heart rate, blood pressure and RPP responses to fast food are illustrated in Fig. 3a-d.

The type 2 diabetes group had an increased heart rate versus controls (*p* < 0.01 or *p* < 0.05) at all time-points except 30 minutes after fast food (*p* = 0.06). Resting heart rate increased within 30 minutes after the meal and subsequently decreased in both groups; it decreased to a larger extent in controls than in type 2 diabetes. Only the controls re-gained baseline resting heart rate after 4 hours (Fig. 3a).

From baseline-1 to baseline-2, systolic blood pressure decreased in type 2 diabetes, but not in controls. Within 2 hours after fast food, the mean systolic blood pressure in the type 2 diabetes group decreased and subsequently reversed within 4 hours post-meal. In contrast, systolic blood pressure in the controls did not change after ingestion of fast food ingestion (Fig. 3b).

Overall, RPP was higher in the type 2 diabetes group versus to controls (baseline-1 and 4 hours, *p* < 0.01; baseline 2 and 2 hours: *p* < 0.05; 30 minutes, *p* = 0.05). The RPP was increased post-meal in both controls and type 2 diabetes (*p* < 0.01 and *p* < 0.05, respectively). It subsequently reduced within 2 hours (*p* < 0.01 and *p* < 0.05, respectively), but was changed back to baseline 2 levels after 4 hours only in the controls (Fig. 3d).

Pre- exercise did not influence heart rate, blood pressure response or RPP (Fig. 3e-h).

### Biochemistry

The response of circulating glucose, C-peptide, triglycerides and TAS to fast food is illustrated in Fig. 4a-d, respectively. Fast food increased the glucose levels within 30 minutes after the meal in both groups. The subsequent drop in mean glucose levels was delayed in the type 2 diabetes group versus the control group. This was indicated by the fact that the difference 2 hours versus 30 minutes post-meal is significant for controls (*p* < 0.001) but not for type 2 diabetes group (*p* = 0.5)— only the controls returned to baseline glucose levels 4 hours post-meal (Fig. 4a). Concurrently, C-peptide levels peaked at 30 minutes post-meal in controls versus at 2 hours post-meal in the type 2 diabetes group (Fig. 4b).

The type 2 diabetes group had indications of an overall higher triglyceride level than the controls (*p* < 0.05, Fig. 4c). The effect of fast food on triglyceride levels was similar in both groups.

The TAS response to fast food was similar for the two groups—TAS decreased within 4 hours in both controls and type 2 diabetes (*p* < 0.01 and *p* < 0.05, respectively; Fig. 4d). Hs-CRP, HDL and LDL did not change in the postprandial phase in either group.

Pre-exercise did not influence any biochemical variables measured at any time-point (Fig. 4e-h).

## Discussion

Our findings indicate that fast-food induces greater overall cardiac workload in type 2 diabetes individuals than in BMI and age matched controls. Pre-exercise did not modify fast food induced changes in LV function, resting heart rate, blood pressure, blood glucose, triglycerides or total antioxidant status.

The observed postprandial increase in diastolic workload in both type 2 diabetes and healthy overweight individuals is novel. Our data contrast the few previous studies that investigated the effects of lipid infusions or a carbohydrate rich meal on cardiac function [9, 10]. Further study is needed to determine whether this is a result of the “combined meal”  used here. The other studies [9, 10] might also have missed an initial increase in diastolic workload due to measuring postprandial response 1 or 2 hours after infusion or ingestion, respectively. In contrast to our finding of increased diastolic workload (e’) after the meal, von Bibra et al. [9] observed a significantly reduced diastolic workload (e’) 2 hours after ingesting a pure carbohydrate meal (48 g). Nielsen et al. [22] found no changes in diastolic function after short-term hyperglycemia by insulin discontinuation in insulin dependent type 2 diabetes individuals. However, these participants [9, 22] had longer history of type 2 diabetes and were insulin dependent.

The present study indicates that fast food interacts with LV diastolic function to a greater extent in type 2 diabetes individuals compared to controls. This could be explained by the prolonged postprandial increase in heart rate in the type 2 diabetes group: An increased heart rate increases diastolic function (E, e’) and shortens IVRT. The decrease in filling pressure in the type 2 diabetes group within 2 h post-meal, could be explained by the indication of sustained increase in e’ at this time point in this group while E is reduced.

We could speculate whether the postprandial diastolic compensations observed in the type 2 diabetes group is an early sign of diastolic dysfunction. However, further research is needed to investigate the progress and interaction of food ingestion and diastolic compensations in type 2 diabetes across different disease stages.

The increased LV systolic workload after fast food ingestion is in line with Holland et al. [10] who demonstrated increased systolic workload (LV global strain rate) induced by increased circulating triglycerides after intra venous administration of a fat emulsion in healthy individuals, and Nielsen et al. [22] who observed increased systolic workload (S’ and strain rate) due to hyperglycemia in type 2 diabetes individuals with and without heart failure. However, our data is in contrast to von Bibra et al. [9] who observed no postprandial change in systolic function (S’) in insulin dependent type 2 diabetes individuals with longer duration after ingesting carbohydrates. The diverse findings may be due to different measurement times as well as differences in methods used to increase circulating glucose and/or triglycerides.

The RPP differences seen here between groups suggest that the type 2 diabetes group had greater cardiac workload compared to controls.

The higher heart rate at rest and prolonged increases in heart rate after fast food consumption by type 2 diabetes individuals relative to controls may be due to several factors including cardiovascular autonomic neuropathy (CAN) that can cause abnormalities in heart rate control by reduced vagal activity and/or high sympathetic activity [23]. Furthermore, both glucose ingestion [24] and elevated plasma fatty acid concentrations [25] may stimulate the cardiac autonomic nervous system with a possible increase in catecholamines. Thus, the effects of fast food seen here may be due to catecholamine induced increases in inotropy that result in increased contractility of the cardiac muscle as well as increased dromotropic and chronotropic effects that increases the heart rate.

Although increased heart rate is commonly observed during euglycemic clamp in this patient group as well as those with metabolic syndrome [23], von Bibra et al. [9] observed no particular increase in resting heart rate 2 hours after a carbohydrate-rich meal in insulin dependent type 2 diabetes individuals. This may be due to the long standing type 2 diabetes [9], which increase the possibility of depressed sympathetic activity [23]. Nielsen et al. [26] observed a tendency towards increased heart rate (*p* = 0.08) due to high levels of lipid infusion versus low lipid infusion controls—this indicates that fast food may increase heart rate more than healthy foods.

The postprandial reduction in systolic blood pressure in the type 2 diabetes group might be an early stage of postprandial systolic hypotension, which is a common hemodynamic condition in diabetes [27] and is associated with an increased risk of cerebrovascular disease [28]. The mechanisms mediating postprandial reductions in blood pressure are not fully understood, but food-induced neurohormonal changes leading to reduced vascular resistance in the splanchnic vasculature as well as CAN, resulting in impaired sympathetic nervous activity has been suggested [29–31].

Gudmundsdottir et al. [32] investigated the postprandial changes after a healthy meal versus a fast food meal and found small changes in blood lipids and hs-CRP with no differences between meals. Our study supports these findings with no changes in cholesterol and hs-CRP.

In the present study, pre-exercise did not modify TAS in type 2 diabetes or controls. This is in contrast to Tyldum et al. [8] who observed a significant exercise-induced improvement in TAS, associated with improvements in endothelial function in healthy normal weight men (42 ± 4 years) using the same protocol as described here. This indicates that our participants had a poorer response to exercise versus lean and healthy individuals [8]. This may possibly be due to central obesity and poorer metabolic control in our study participants versus normal weight individuals.

The lack of exercise-induced improvements in postprandial TAS in the present study may be explained by a lack of exercise induced postprandial changes in circulating glucose- and triglyceride levels. Although the lack of exercise-induced TAS changes contrast with the findings of Tyldum et al. [8], the lack of exercise induced changes in glucose- and triglyceride levels did concur. Nevertheless, exercise-induced changes have previously been observed due to acute exercise on postprandial triglyceride levels and hyperglycemia [33–35].

However, studies are difficult to compare due to different measurement methods, meal composition and size, timing of exercise, exercise mode, intensity and duration. Inadequate energy expenditure [36] and/or inadequate exercise timing relative to the meal may explain the lack of exercise induced reduction in postprandial glucose [37] - and/or triglyceride excursion [38], etc. The acute effect of different exercise modes and timing of these on the postprandial response of the LV certainly needs to be further investigated in type 2 diabetes as the time course of adaptation may be different in the heart/endothelium than normal body weight persons.

## Conclusions

Our findings indicate that fast food induces greater and sustained overall cardiac workload in the postprandial phase in type 2 diabetes individuals compared to BMI and age matched controls. Pre-exercise had no acute effects to the postprandial phase. The acute interaction of food on cardiac function in type 2 diabetes needs further study. More research is also needed on the effects of other exercise methods and exercise timing on postprandial cardiac function and other cardiovascular risk markers in this patient group.

### Limitations

The limitations of this study include a small sample size and similarity (BMI and WC) between groups. We evaluated the effect of fast-food on LV function, and therefore the individual effects of carbohydrates, fat and salt cannot be evaluated. A recent study demonstrated no effect on diastolic function in normotensive healthy men after one week of high dietary sodium intake [39]. However a previous study found that one week of high dietary sodium intake impair myocardial relaxation [40]. The strengths of this study include the strictly controlled study environment, supervised exercise interventions as well as the similarity in BMI and WC between groups as it excludes potentially confounding effects of adiposity.
